# Systematic pan-cancer analysis of the potential tumor diagnosis and prognosis biomarker P4HA3

**DOI:** 10.3389/fgene.2023.1045061

**Published:** 2023-03-22

**Authors:** Yinteng Wu, Bo Zhang, Juan Nong, Raquel Alarcòn Rodrìguez, Wenliang Guo, Ying Liu, Shijian Zhao, Ruqiong Wei

**Affiliations:** ^1^ Department of Orthopedic and Trauma Surgery, The First Affiliated Hospital of Guangxi Medical University, Nanning, Guangxi, China; ^2^ Department of Trauma Hand Surgery, The Second Nanning People’s Hospital, Nanning, Guangxi, China; ^3^ Department of Joint Surgery, The Second Nanning People’s Hospital, Nanning, Guangxi, China; ^4^ Faculty of Health Sciences, University of Almerìa, Almeria, Spain; ^5^ Department of Rehabilitation Medicine, Guigang City People’s Hospital, Guigang, China; ^6^ Department of Rehabilitation Medicine, The First Affiliated Hospital of Guangxi Medical University, Nanning, Guangxi, China; ^7^ Department of Cardiology, The Affiliated Cardiovascular Hospital of Kunming Medical University (Fuwai Yunnan Cardiovascular Hospital), Kunming, Yunnan, China

**Keywords:** prolyl 4-hydroxylase subunit alpha 3 (P4HA3), pan-cancer, the tumor microenvironment (TME), collagen, immunotherapy, epithelial-mesenchymal transition (EMT)

## Abstract

**Purpose:** Prolyl 4-hydroxylase subunit alpha 3 (*P4HA3*) is implicated in several cancers’ development. However, *P4HA3* has not been reported in other cancers, and the exact mechanism of action is currently unknown.

**Materials and methods:** First, the expression profile of *P4HA3* was analyzed using a combination of the University of California Santa Cruz (UCSC) database, Cancer Cell Line Encyclopedia (CCLE) database, and Genotype-Tissue Expression (GTEx) database. UniCox and Kaplan-Meier were used to analyze the predictive value of *P4HA3*. The expression of *P4HA3* was analyzed in clinical staging, immune subtypes, and Molecular subtypes. Secondly, the correlation of *P4HA3* with immunomodulatory genes, immune checkpoint genes, RNA modification genes, immune cell infiltration, cancer-related functional status, tumor stemness index, DNA mismatch repair (MMR) genes and DNA Methyltransferase was examined. The role of *P4HA3* in DNA methylation, copy number variation (CNV), mutational status, tumor mutational burden (TMB), and microsatellite instability (MSI) was also analyzed. In addition, gene set enrichment analysis (GSEA) was used to explore the potential functional mechanisms of *P4HA3* in pan-cancer. Finally, *P4HA3*-related drugs were searched in CellMiner, Genomics of Drug Sensitivity in Cancer (GDSC), and Cancer Therapeutics Response Portal (CTRP) databases.

**Results**: *P4HA3* is significantly overexpressed in most cancers and is associated with poor prognosis. *P4HA3* is strongly associated with clinical cancer stage, immune subtypes, molecular subtypes, immune regulatory genes, immune checkpoint genes, RNA modifier genes, immune cell infiltration, cancer-related functional status, tumor stemness index, MMR Gene, DNA Methyltransferase, DNA methylation, CNV, mutational status, TMB, and MSI are closely related. Available enrichment analysis revealed that *P4HA3* is associated with the epithelial-mesenchymal transition and immune-related pathways. There are currently 20 drugs associated with *P4HA3*.

**Conclusion**: In human pan-cancer, *P4HA3* is associated with poor patient prognosis and multiple immune cells and may be a novel immunotherapeutic target. It may act on tumor progression through the epithelial-mesenchymal transition (EMT) pathway.

## Introduction

According to the latest data on cancer from the 2020 statistics, the worldwide cancer burden is expected to increase by 47% by 2040 compared to 2020 (Sung et al., 2021). Cancer immunotherapy is a treatment that fights cancer by boosting the patient’s immune system. Immunotherapy, a vital component of antitumor therapy, is not meeting the needs of the growing number of cancer patients, despite the continuous updating of its treatments. Therefore, searching for new and particular molecular markers of cancer is crucial for developing more effective anti-cancer drugs. The pan-cancer analysis is known to reveal the potential molecular mechanisms and functional roles among cancers systematically and comprehensively (Weinstein et al., 2013). In recent years, pan-cancer-based bioinformatics analysis methods have been highly favored by a wide range of researchers.

The extracellular matrix (ECM), the most abundant component of the tumor microenvironment (TME), plays a crucial role in tumor development (Bonnans et al., 2014; Xiong and Xu, 2016). Collagen is one of the significant components of the ECM and is associated with cancer progression (Armstrong et al., 2004; Koenig et al., 2006; Shintani et al., 2008; Gorres and Raines, 2010; Yang et al., 2014; Peng et al., 2017; Zheng et al., 2017; Yamazaki et al., 2018). It has been shown that cancer cells produce a type 1 collagen (Col I) that is fundamentally different from normal collagen in humans, resulting in a unique extracellular matrix that helps them increase and survive (Chen et al., 2022). Previous studies have found that Col I promotes cancer progression by facilitating tumor cell epithelial-mesenchymal transition (EMT) (Yang et al., 2014). Recent studies have found that loss of Col I in pancreatic cancer enhances T-cell infiltration, thus making anti-PD-1 immunotherapy more effective (Chen et al., 2022). Collagen deposition leads to poor prognosis in cancer patients (Whatcott et al., 2015). In addition, it is involved in tumor fibrosis (Yamauchi et al., 2018). Collagen prolyl 4-hydroxylases (C-P4H) are critical enzymes in collagen synthesis that maintain the triple helix structure’s stability by catalyzing the proline’s hydroxylation (Gjaltema and Bank, 2017). Interfering with prolyl 4-hydroxylase can regulate the process of collagen biosynthesis (Atkinson et al., 2019; D'Aniello et al., 2019). Recent studies have revealed that C-P4H is expressed at elevated levels in several cancers and may be a potential biomarker (Vasta and Raines, 2018). C-P4H is a tetramer composed of two *a*-subunits and two *ß*-subunits. Among them, the catalytic *a*-subunit has three different isozymes such as *P4HA1*, *P4HA2*, and *P4HA3* (Vuori et al., 1992; Helaakoski et al., 1995; Van Den Diepstraten et al., 2003).

*P4HA3* (prolyl 4-hydroxylase subunit alpha 3) is a protein-coding gene that belongs to the prolyl 4-hydroxylase family members. A study found that *P4HA3* expression is upregulated in patients with renal cell carcinoma (RCC), promoting cancer growth, invasion, and metastasis (Zhou et al., 2022a). It has been reported that in gastric cancer (GC), *P4HA3* is significantly upregulated compared to normal tissue and is associated with unfavorable overall survival (OS) (Song et al., 2018). *P4HA3* is located upstream of the PI3K/AKT signaling pathway, and type VI α6 collagen (COL6A6) interacts with *P4HA3* to inhibit pituitary adenoma (PA) growth and metastasis by blocking the PI3K/AKT signaling pathway (Long et al., 2019). In addition, it was found that *P4HA3* overexpression on the PI3K/AKT signaling pathway reversed the inhibitory effect of COL6A6 on EMT (25). *P4HA3* is overexpressed in melanoma and promotes tumor proliferation and invasion (Atkinson et al., 2019). The above findings suggest that *P4HA3* is overexpressed in various cancers and is involved in cancer progression. However, its pan-cancer expression level and functional role have not been explored.

In this study, we evaluated the expression of P4HA3 and its relationship with the prognosis of cancer patients. In addition, the relationship between P4HA3 and the tumor immune microenvironment was further analyzed. Our findings provide new insights into the functional role of P4HA3 in human cancer, highlighting the potential mechanisms by which P4HA3 affects the tumor microenvironment and cancer immunotherapy.

## Materials and methods

### Collection and analysis of *P4HA3* expression data

RNA-seq and clinical data of The Cancer Genome Atlas (TCGA) were downloaded from the UCSC Xena database (https://xenabrowser. net/datapages/). The UCSC database includes expression matrices and clinical data of pretreated TCGA tumor patients and normal subjects, including overall survival (OS), disease-specific survival (DSS), disease-free interval (DFI), and progression-free interval (PFI), tumor stage, *etc.* First, *P4HA3* expression data of normal tissues were obtained from the GenotypeTIssup Expression (GTEx, https://commonfund.nih.gov/GTEx) database. And *P4HA3* expression data of tumor cell lines were obtained from the Cancer Cell Line Encyclopedia (CCLE, https://portals.broadinstitute.org/ccle/) database. Second, a uniformly normalized pan-cancer dataset was obtained from the UCSC Xena database to analyze the expression levels of *P4HA3* in cancer and the differential expression in cancer and normal samples. Meanwhile, we analyzed the expression of *P4HA3* in male and female tumor tissues and the corresponding normal tissues using the R package “gganatogram” (Maag, 2018) and visualized it as BodyMap. We analyzed the protein expression level of *P4HA3* using The Human Protein Atlas (HPA) database (https://www.proteinatlas.org). Many researchers have used this database to analyze the expression levels of target genes, such as Xiong et al., Zhou et al., Liu et al., and Xu et al. used this database to study the expression levels of PIMREG, NAAA, CD2, and TUBA1C respectively, which tentatively proved their analysis results (Chen et al., 2021; Huang et al., 2021; Zhu et al., 2021; Hu et al., 2022).

### Survival and prognostic analysis of *P4HA3*

To understand the impact of *P4HA3* on the survival and prognosis of cancer patients, we used Kaplan-Meier curve analysis and univariate Cox proportional hazard regression to explore the relationship between *P4HA3* expression levels, and OS, DSS, DFI, and PFI. A *p*-value <0.05 was statistically significant.

### Analysis of *P4HA3* expression in tumor clinical stage, immune subtypes, and molecular subtype

The expression levels of *P4HA3* were assessed in each tumor at different clinical stages. Next, the immune subtypes of *P4HA3* in 33 tumors, including C1 (wound healing), C2(IFN-gamma dominant), C3 (inflammatory), C4 (lymphocyte depleted), C5 (immunologically quiet), and C6 (TGF-b dominant), were obtained in the TISIDB database (https://doi.org/10.1093/bioinformatics/btz210). In addition, the molecular subtype profile of *P4HA3* in 13 tumors was also analyzed. We analyzed the association between P4HA3, immune, and molecular subtypes through TISIDB database of pre-processed gene expression data and phenotypic information of TCGA cancers.

### Correlation analysis of *P4HA3* with immunomodulatory genes, immune checkpoint genes, and RNA-modified genes

The expression data of 150 marker genes for immune regulation (chemokine (Qiu et al., 2019), receptor (D'Aniello et al., 2019), MHC (Helaakoski et al., 1995), Immunoinhibitor (Song et al., 2018), Immunostimulator (Chae et al., 2019)), 60 marker genes for immune checkpoint-related genes (Inhibitory (Song et al., 2018), Stimulatory (Mariathasan et al., 2018)) and 44 marker genes for RNA modification (m1A (Yamazaki et al., 2018), m5C (Chen et al., 2022), m6A (Helaakoski et al., 1995)) genes were extracted in each sample. Then, the correlations between *P4HA3* and immune regulatory genes, immune checkpoint genes, and RNA modification genes were calculated separately using the spearman algorithm.

### Analysis of tumor immune cell infiltration

The correlation between *P4HA3* and many different types of immune cells was analyzed in the TIMER2.0 database (https://cistrome.shinyapps.io/timer/) using various algorithms such as TIMER, CIBERSORT, CIBERSORT-ABS, QUANTISEQ, XCELL, EPIC, and TIDE. The TIMER 2.0 database provides a more reliable analysis of target gene and immune cell correlations by using state-of-the-art algorithms to calculate the level of immune infiltration in TCGA tumor data. The results were visualized using the R package “ggplot2” (Ito and Murphy, 2013). In addition, we used the ESTIMATE algorithm to calculate the ESTIMATEScore, ImmuneScore, and StromalScore for different tumor types and the spearman algorithm to calculate the correlation coefficient between *P4HA3* and the three scores.

### Correlation analysis of *P4HA3* with single cell level, cancer-related functional status, and tumor stemness index

We obtained 72 single-cell datasets from the CancerSEA database (https://ngdc.cncb.ac.cn/databasecommons/database/id/6092) as well as 14 cancer-related functional states (angiogenesis, apoptosis, cell cycle, cell differentiation, DNA damage, DNA repair, EMT, cellular hypoxia, inflammation onset, cancer cell invasion, metastasis, proliferation cell resting, stem cell properties). The correlation between *P4HA3* and 14 cancer-related functional states in each tumor was analyzed and visualized using the R package “ggplot2”. In addition, we obtained DNAss tumor stemness scores calculated from methylation profiles for each cancer from a previous study (Malta et al., 2018). The stemness index and *P4HA3* expression data of the samples were first integrated, then the correlation between them was calculated using the spearman algorithm. To further validate our results, tumor stemness scores such as EREG-METHss, DMPss, and ENHss were also performed.

### Evaluation of *P4HA3* mutation, methylation, TMB, MSI, MMR gene, DNA methyltransferase, and CNV in pancreatic cancer

To understand the mutation characteristics and location of *P4HA3* in tumors, we explored using the cBioPortal database (https://www.cbioportal.org/). Meanwhile, using the TCGA database (https://www.cancer.gov/about-nci/organization/ccg/research/structural-genomics/tcga) we analyzed the relationship between the expression level of *P4HA3* and the methylation level of its promoter region and visualized it using the R package “ggplot2”. The correlation between *P4HA3* expression and tumor mutational burden (TMB) and microsatellite instability (MSI) in different tumors was analyzed in the TCGA database using Spearman’s test, and the results were visualized by the R package “fmsb”. In addition, we also analyzed the correlation between *P4HA3* and five DNA mismatch repair (MMR) genes, MLH1, MSH2, MSH6, PMS2, and EpCAM. and their expression levels with the four methyltransferases DNMAT1, DNMT2, DNMT3A, and DNMT3B. Not only that, but we also analyzed the correlation between *P4HA3* expression and somatic cell copy number variation (CNV) in pan-cancer.

### Enrichment analysis of *P4HA3*

To further understand the biological functions and potential molecular mechanisms of *P4HA3*, we used the R package “ClusterProfiler” (Yu et al., 2012) to perform gene set enrichment analysis (GSEA). We also downloaded from the Molecular Signatures Database (MSigDB, https://www.gseamsigdb.org/gsea/index.jsp) a hallmark gene set containing 50 critical pathways affecting cancer. The Normalized Enrichment Score (NES) and False Discovery Rate (FDR) of *P4HA3* in each path were calculated separately. *p*-values <0.05 were considered statistically significant.

### Drug sensitivity analysis

To understand the potential drugs targeting *P4HA3*, we analyzed the relationship between *P4HA3* expression and drug sensitivity using the CellMiner database (https://ngdc.cncb.ac.cn/databasecommons/database/id/6092). Meanwhile, to further expand our study, we also explored the correlation between *P4HA3* expression and drug sensitivity from the Genomics of Drug Sensitivity in Cancer (GDSC) database (https://www.cancerrxgene.org) and Cancer Therapeutics Response Portal (CTRP) database (https://portals.broadinstitute.org/ctrp.v2.1/).

## Results

### Expression profile of *P4HA3*

**FIGURE 1:**
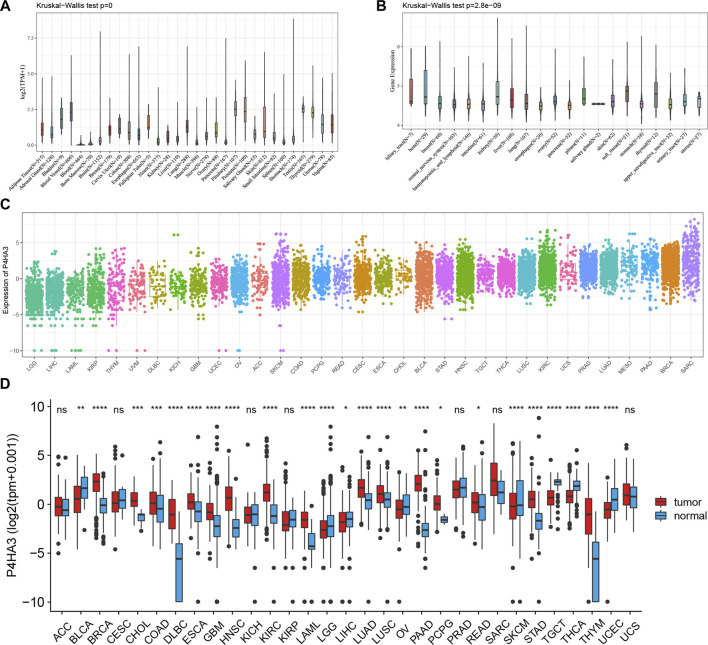
Expression of *P4HA3* in normal and tumor tissues. **(A)** *P4HA3* expression across 31 regular tissues and **(B)** 21 tumor cell lines. The mRNA expression landscape of *P4HA3* in **(C)** tumor tissue on TCGA database and **(D)** expression of *P4HA3* in normal and tumor tissues (*p < 0.05, **p < 0.01, and ***p < 0.001; ns: no significance). *p* values were based on the Wilcoxon rank sum test.

**FIGURE 2:**
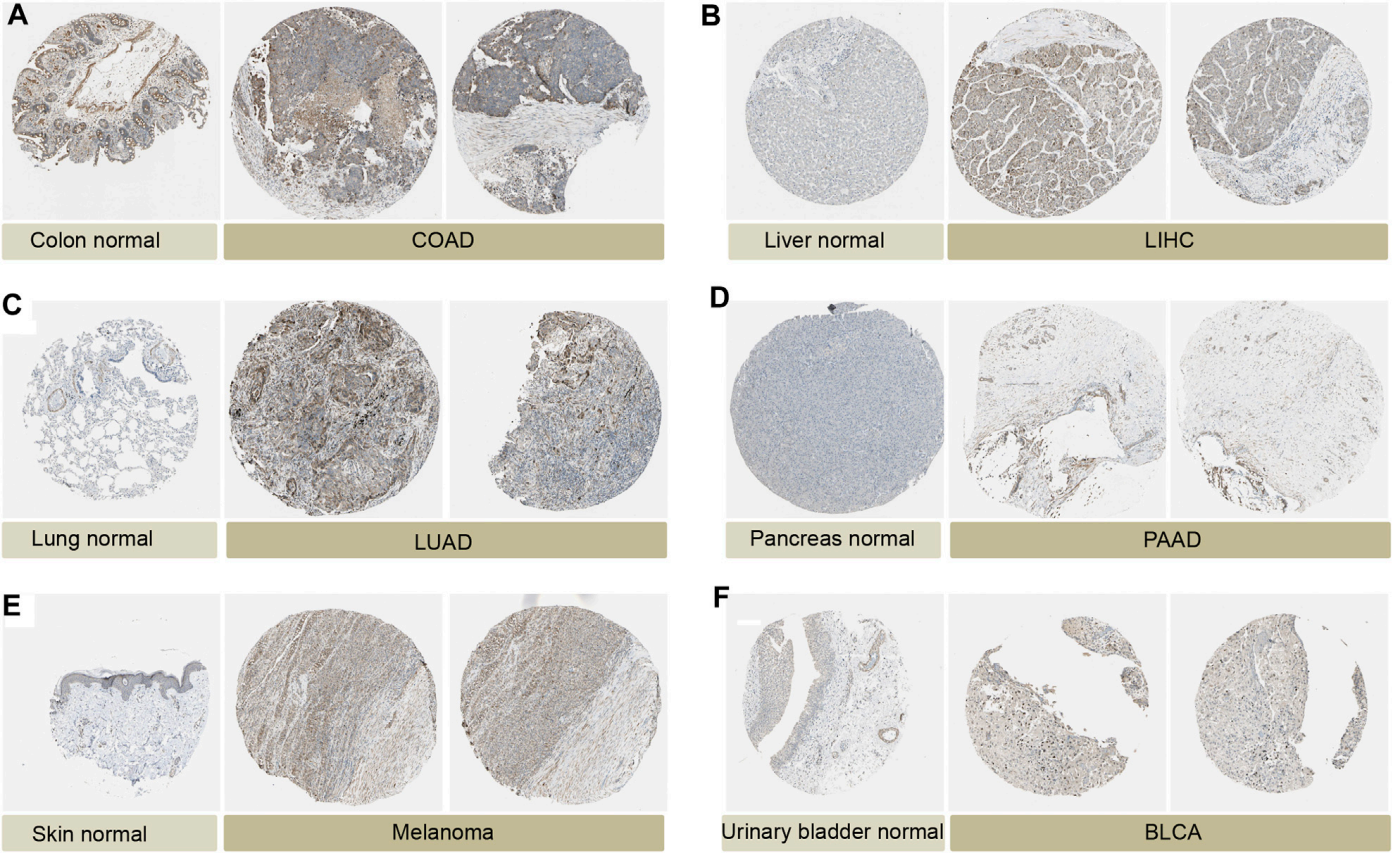
Protein expression of *P4HA3* in normal and tumor tissues. Immunohistochemical staining in normal tissues and tumour tissues from the HPA database. **(A)** Colon normal, COAD. **(B)** Liver normal, LIHC. **(C)** Lung normal, LUAD. **(D)** Pancreas normal, GBM. **(E)** Skin normal, Melanoma. **(F)** Urinary bladder normal, BLCA.

We analyzed the expression levels of *P4HA3* in normal tissues using the GTEx dataset (Figure 1A). The results showed that *P4HA3* expression levels were higher in testis, pituitary, prostate, blood vessels, and thyroid. Based on the CCLE dataset, the expression levels of *P4HA3* in different tumor cell lines were analyzed (Figure 1B). The results showed that the expression of *P4HA3* was relatively similar in various tumor cell lines. In addition, the expression levels of *P4HA3* in different tumors were shown in Figure 1C. Data from the TCGA database and GTEx database showed that *P4HA3* was expressed in breast invasive carcinoma (BRCA), cholangiocarcinoma (CHOL), colon adenocarcinoma (COAD), lymphoid neoplasm diffuse large B cell lymphoma (DLBC), esophageal carcinoma (ESCA), glioblastoma (GBM), head and neck squamous cell carcinoma (HNSC), lung adenocarcinoma (LUAD), kidney renal clear cell carcinoma (KIRC), acute myeloid leukemia (LAML), lung squamous cell carcinoma (LUSC), pancreatic adenocarcinoma (PAAD), pheochromocytoma and paraganglioma (PCPG), rectum adenocarcinoma (READ), stomach adenocarcinoma (STAD), and thymoma (THYM) were expressed at higher levels than normal tissues in 16 tumors. In contrast, *P4HA3* was expressed at lower levels than normal tissues in eight tumors, including liver hepatocellular carcinoma (LIHC), ovarian serous cystadenocarcinoma (OV), skin cutaneous melanoma (SKCM), thyroid carcinoma (THCA), uterine corpus endometrial carcinoma (UCEC), bladder urothelial carcinoma (BLCA), brain lower grade glioma (LGG), and testicular germ cell tumors (TGCT) (Figure 1D). Finally, we analyzed the expression levels of *P4HA3* in active body maps using the GEPIA dataset. The results show that *P4HA3* was differentially expressed in tumor tissues and corresponding normal tissues, especially in the brain, lung, esophagus, liver, gallbladder, stomach, intestine, thyroid, bladder, and bone (Supplementary Figures 1A, B). The IHC results of COAD, LIHC, LUAD, PAAD, melanoma, BLCA and normal tissues were show in Figure 2. More samples and experiments are needed to validate the expression of P4HA3 in the future. The above results suggest that *P4HA3* is highly expressed in various tumors.

### Survival and prognostic value of *P4HA3*

**FIGURE 3:**
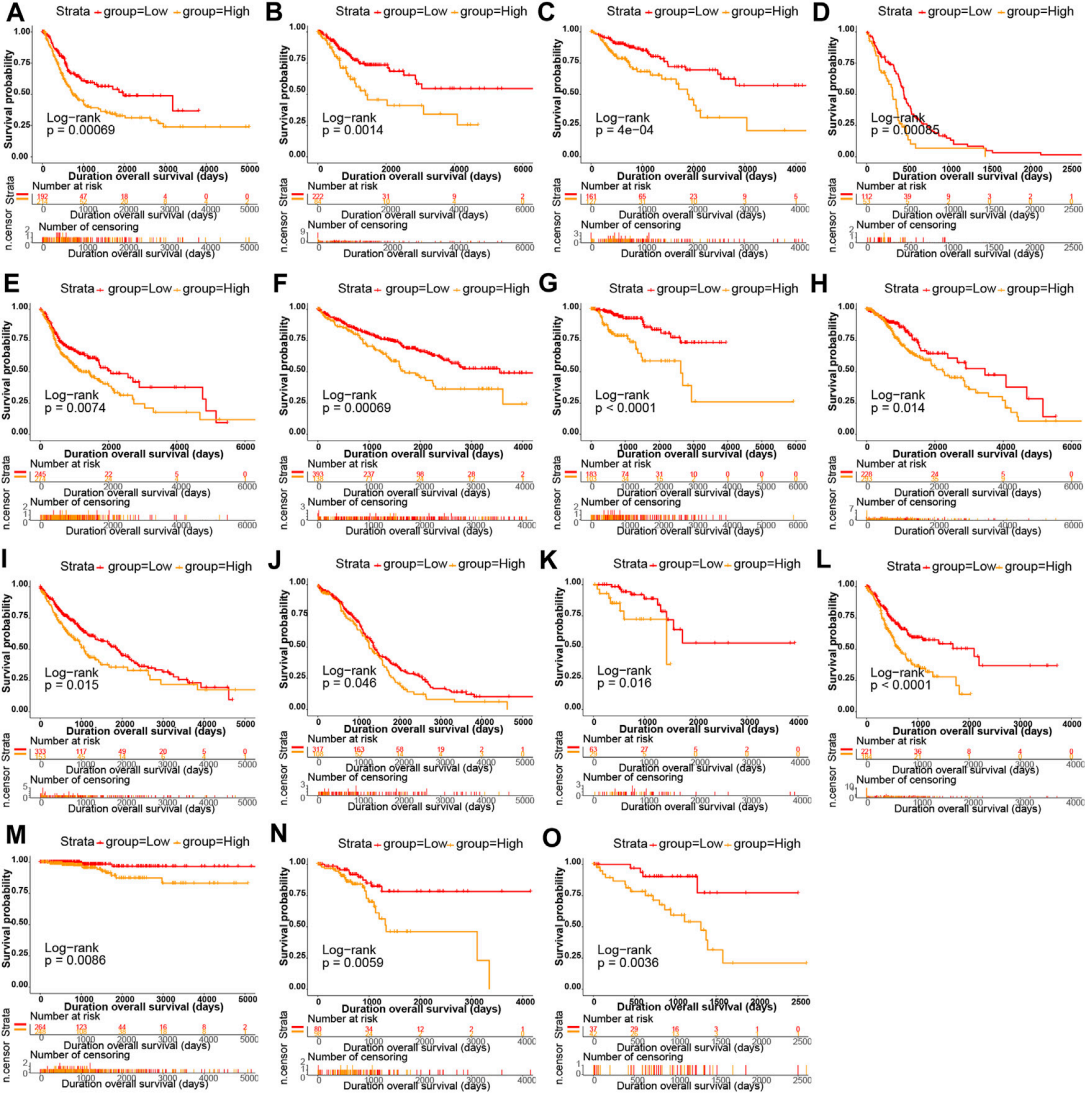
The relationship of *P4HA3* expression with patients’ overall survival (OS). Kaplan–Meier survival analysis of OS between high- and low-expression groups of *P4HA3* in **(A)** ACC **(B)** BRCA, **(C)** CESC **(D)** CHOL, **(E)** GBM **(F)** HNSC, **(G)** KICH **(H)** KIRC, **(I)** LAML **(J)** LGG, **(K)** LIHC **(L)** LUAD, **(M)** LUNG **(N)** MESO, and **(O)** LAML.

**FIGURE 4:**
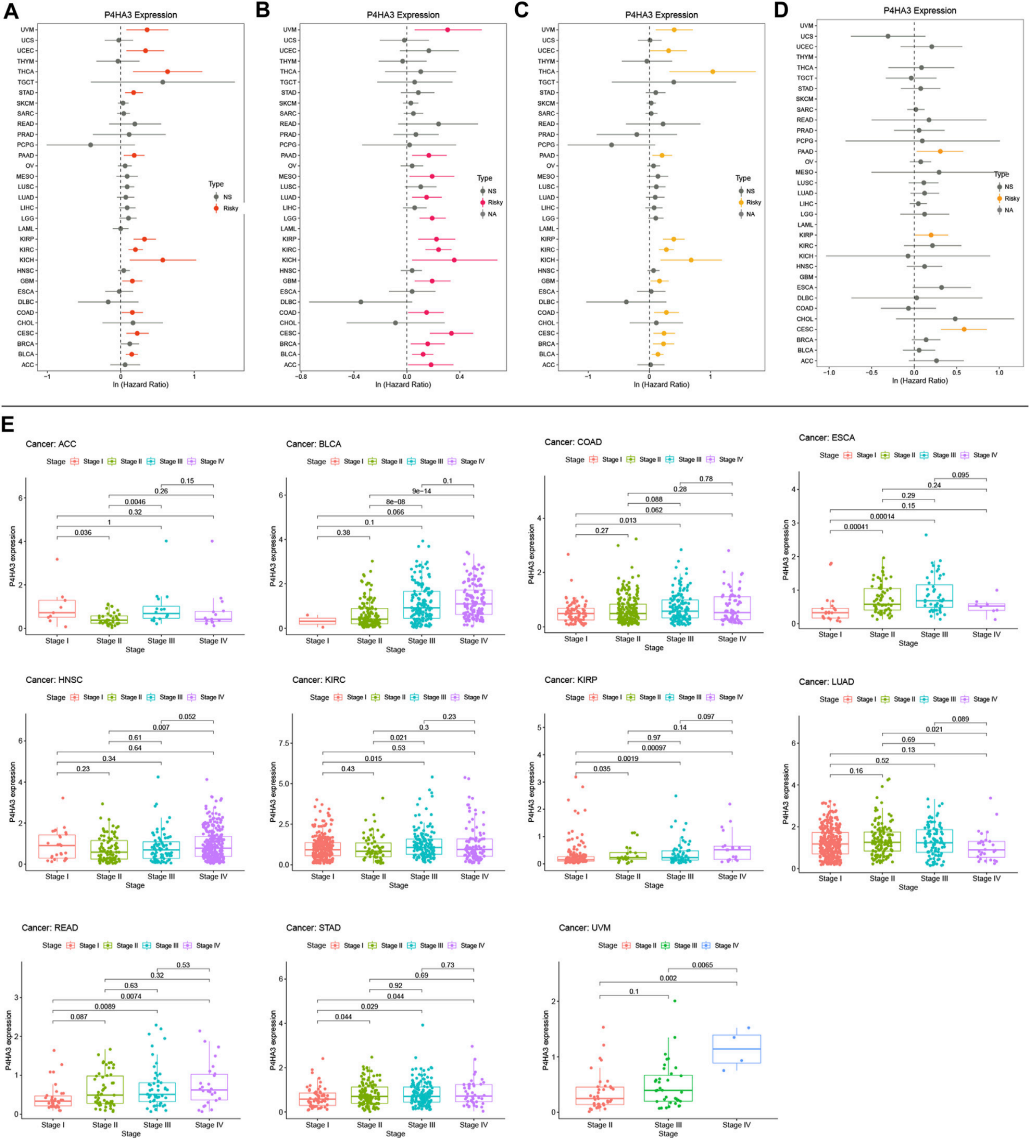
Correlation between *P4HA3* expression with prognosis and clinical stage in cancer patients. Forest plots of hazard ratios of *P4HA3* in **(A)** overall survival (OS) **(B)** progression-free interval (PFI) **(C)** disease-specific survival (DSS), and **(D)** disease-free interval (DFI). Red and yellow indicates that *P4HA3* is a risk factor for the prognosis of cancer patients, while grey represents a protective factor. **(E)** The difference of *P4HA3* expression level between distinct clinical stages in ACC, BLCA, COAD, ESCA, HNSC, KIRC, KIRP, LUAD, READ, STAD, and UVM.

Kaplan-Meier survival analysis showed that *P4HA3* high expression in BRCA, CESC, COAD, GBM, HNSC, KIRC, kidney renal papillary cell carcinoma (KIRP), LGG, LUSC, OV, READ, STAD, THCA, UCEC, and uveal melanoma (UVM) had a poor prognosis (Figures 3A–O). Univariate COX regression analysis assessed the relationship between *P4HA3* expression and OS, DSS, DFI, and PFI. The results showed that *P4HA3* was a risk factor for OS in UVM, UCEC, THCA, STAD, PAAD, KIRP, KIRC, kidney chromophobe (KICH), GBM, COAD, cervical squamous cell carcinoma (CESC), and BLCA (Figure 4A); *P4HA3* was a risk factor for DSS in UVM, PAAD, mesothelioma (MESO), LUAD, LGG, KIRP, KIRC, KICH, GBM, COAD, CESC, BRCA, BLCA, and adrenocortical carcinoma (ACC) (Figure 4B); *P4HA3* was a risk factor for DFI in UVM, UCEC, THCA, PAAD, KIRP, KIRC, KICH, GBM, COAD, CESC, BRCA, and BLCA (Figure 4C); *P4HA3* was a risk for PFI in PAAD, KIRP, and CESC (Figure 4D). The survival analysis results suggest that overexpression of *P4HA3* is associated with poor prognosis in tumor patients.

### Expression of *P4HA3* in different clinical stages of tumors, immune subtypes, and molecular subtypes

**FIGURE 5:**
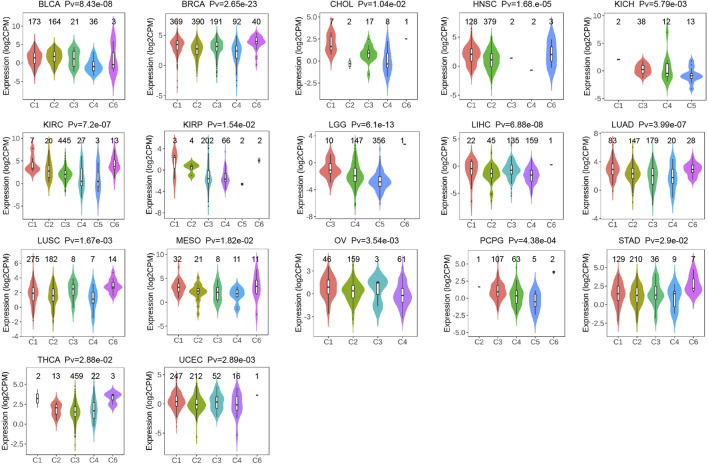
The relationship of *P4HA3* expression with immune subtypes in BLCA, BRCA, CHOL, HNSC, KICH, KIRC, KIRP, LGG, LIHC, LUAD, LUSC, MESO, OV, PCPG, STAD, THCA, and UCEC. The different colors represent the different immune subtypes.

**FIGURE 6:**
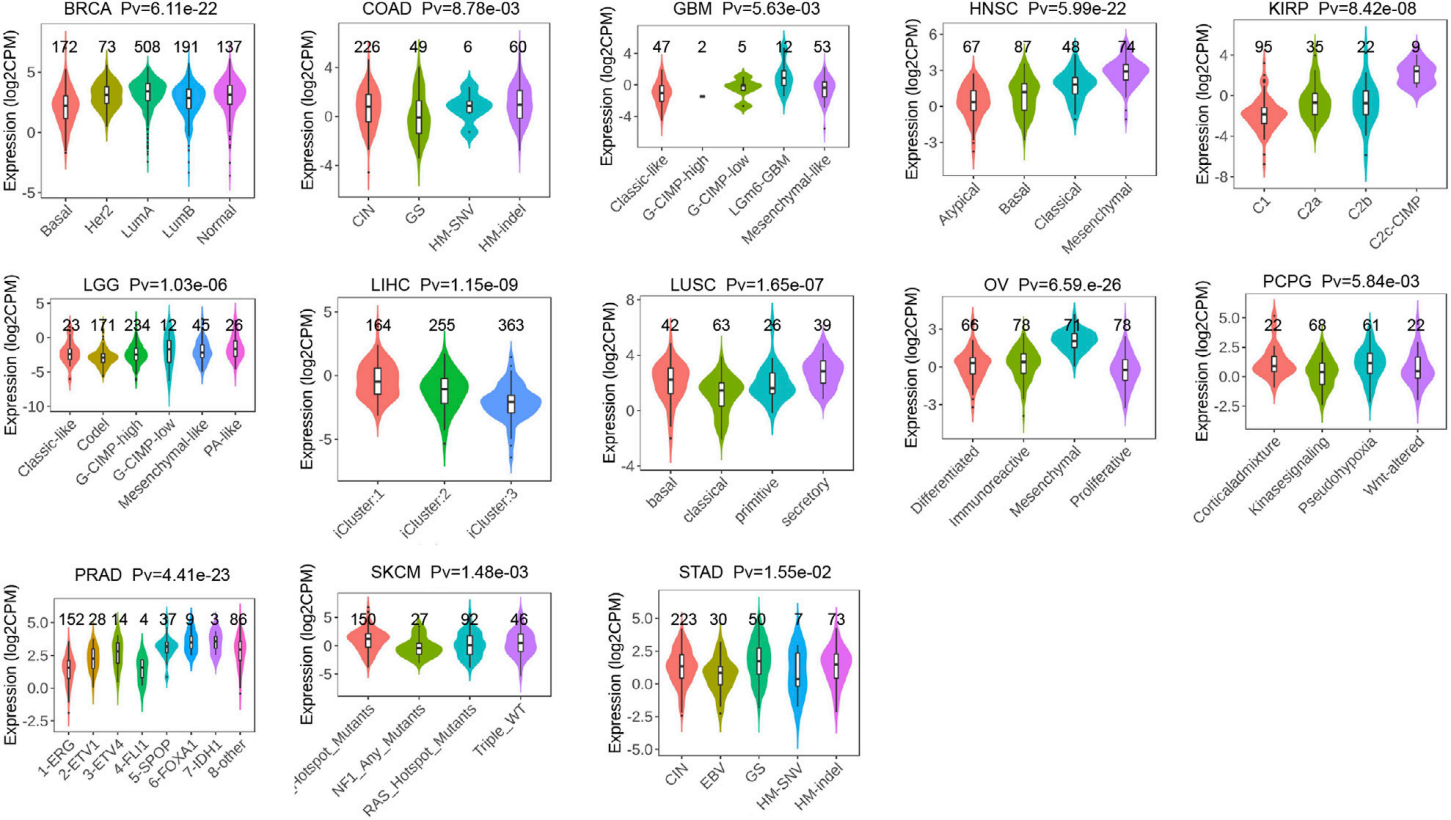
The differences of *P4HA3* expression levels among distinctive molecular subtypes in BLCA, COAD, GBM, HNSC, KIRP, LGG, LIHC, LUSC, OV, PCPG, PRAD, SKCM, and STAD. The different colors represent the different molecular subtypes.

After analysis and evaluation, we found that, in terms of clinical staging, *P4HA3* was more highly expressed at higher stages of BLCA, COAD, ESCA, KIRP, READ, STAD, and UVM. In comparison, lower expression in higher stages of ACC, HNSC, KIRC, and LUAD (Figure 4E). Regarding immune subtypes, *P4HA3* was significantly expressed in 17 tumors (Figure 5). Regarding the molecular subtypes, *P4HA3* was expressed considerably in 13 tumors (Figure 6). Thus, the expression level of *P4HA3* correlated with the clinicopathological stage, immune subtypes, and molecular subtypes of cancer.

### Correlation analysis of *P4HA3* with immunomodulatory genes, immune checkpoint genes, and RNA-modified genes

**FIGURE 7:**
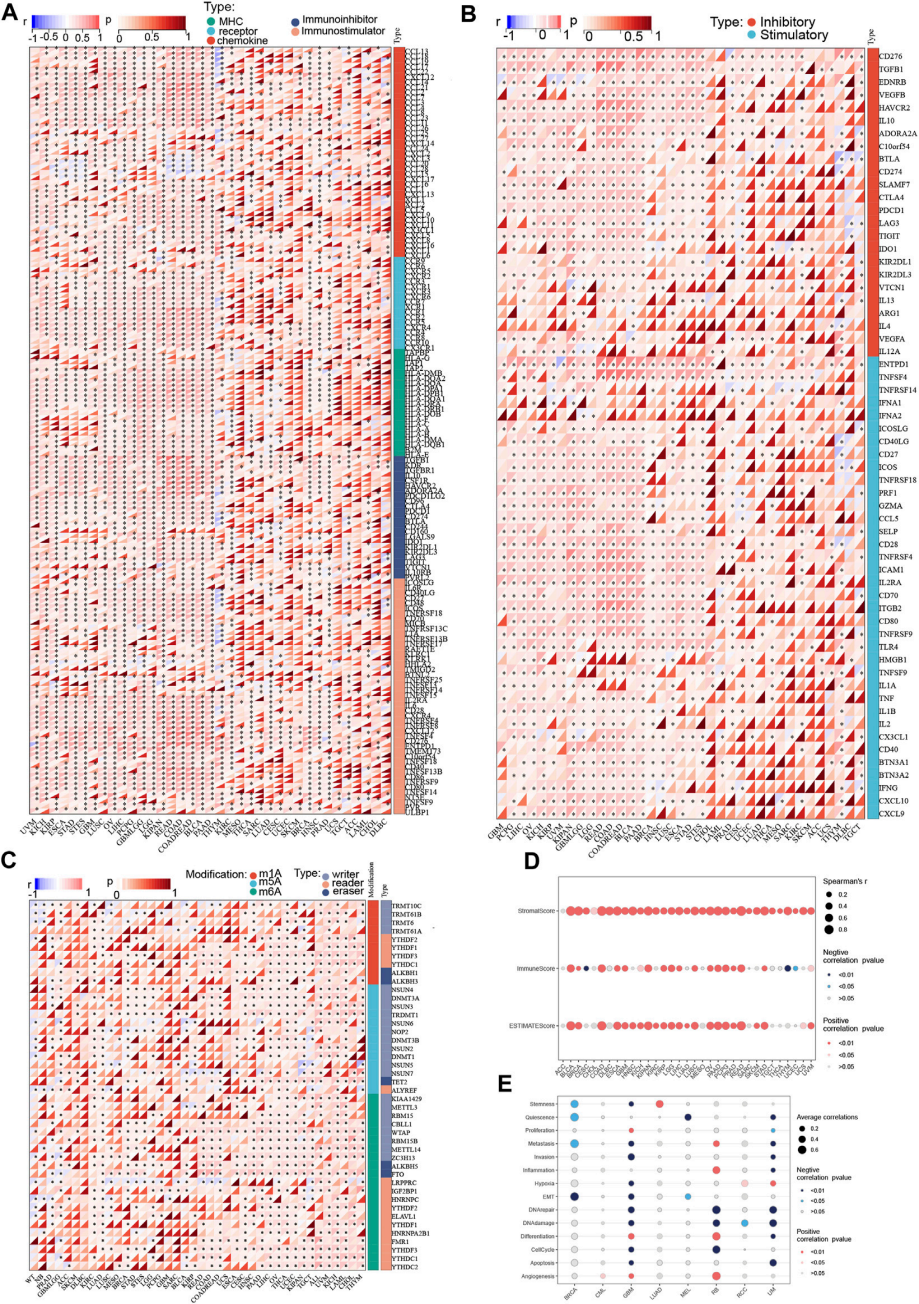
Co-expression of *P4HA3* with immune-associated genes. Co-expression between *P4HA3* and **(A)** Immunoregulatory genes **(B)** Immune checkpoint genes, and **(C)** RNA modifier genes (*p < 0.05, **p < 0.01, and ***p < 0.001). The Relationship of *P4HA3* expression with **(D)** immune infiltration and **(E)** cancer related functional status analysis.

It was found that in most tumors, *P4HA3* was associated with CCL13, CCL18, CXCL12, CCL14, CCL21, CCL2, CCL7, CCL11, CCL26, CXCL3, CXCL8, CCR3, CXCR1, CCR7, CCR1, CXCR4, CCR4, CCR8, CCR10, HLA -DOA, HLA-DPA1, HLA-DPB1, HLA-DRA, B2M, TGFBR1, KDR, TGFBR1, IL10, ADORA2A, CD244, IL2RA, IL6, CD28, CXCR4, TNFRSF4, and CXCL12 immunoregulatory genes were significantly and positively correlated (Figure 7A). It also correlated with immune checkpoint TGFB1, C10orf54, CD276, VEGFA, EDNRB, CTLA4, PDCD1, ENTPD1, TNFSF4, SELP, CD28, TNFRSF4, ICAM1, IL2RE, TNFRSF9, TLR4, IL1B, CX3CL1, and CXCL10 genes were significantly and positively correlated (Figure 7B). In addition, we found that *P4HA3* was significantly associated with genes related to m1A, m5c, and m6A in most tumors (Figure 7C). In most tumors, *P4HA3* positively correlated with immunomodulatory, immune checkpoint, and RNA-modified genes.

### Correlation analysis of *P4HA3* with tumor immune cell infiltration and cancer-related functional status

**FIGURE 8:**
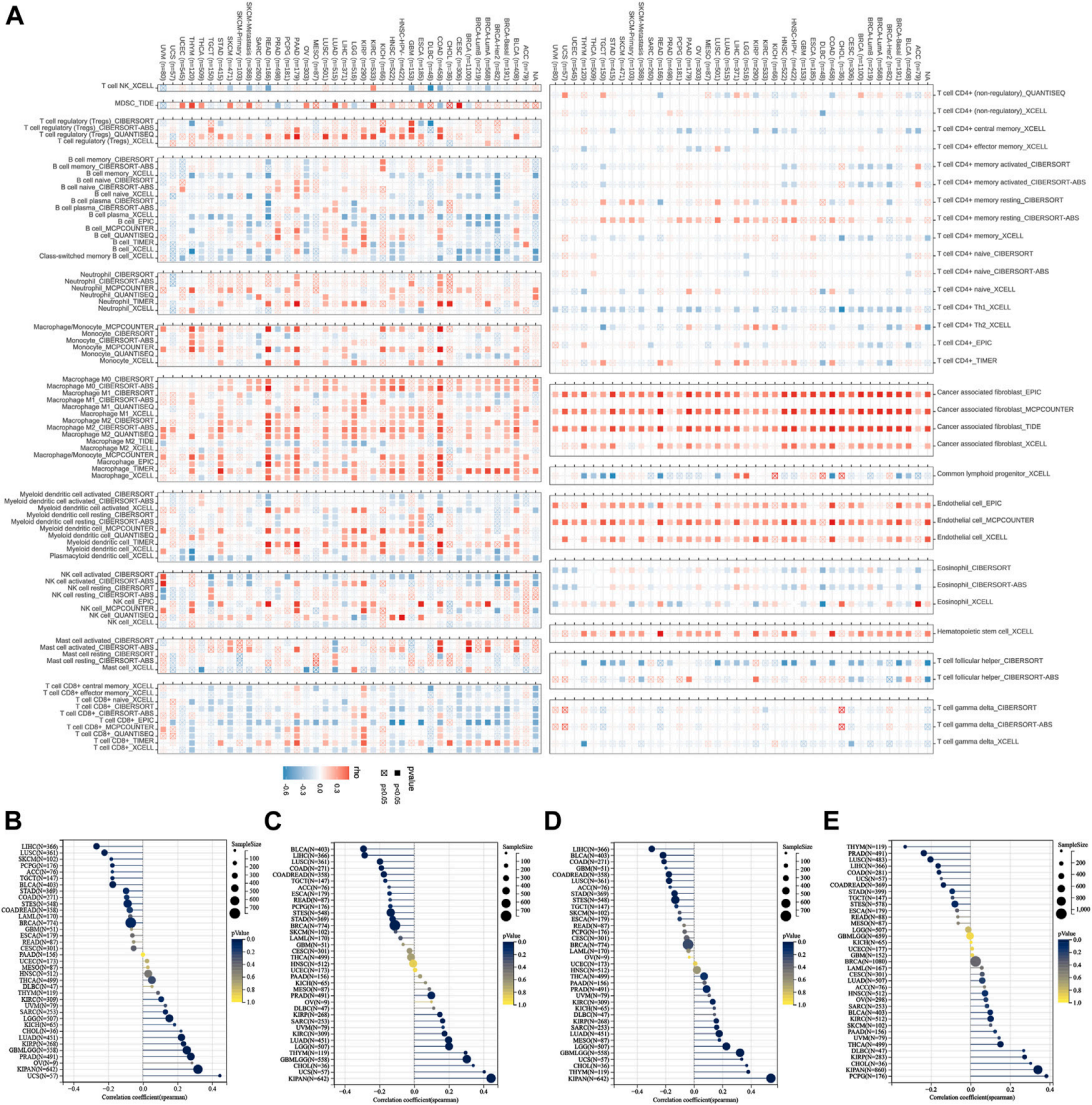
The correlations of *P4HA3* expression and the infiltration levels of CD4^+^ T cells, cancer-associated fibroblast, macrophages, and many other immune cells in cancers **(A)**. Positive correlation in red and negative correlation in blue. The Relationship of *P4HA3* expression with tumor stemness index from **(B)** DMPss **(C)** DNAss, **(D)** ENHss, and **(E)** EREG-METHss algorithm.

The results calculated according to the ESTIMATE algorithm showed that *P4HA3* in different tumor types such as BLCA, BRCA, COAD, ESCA, GBM, KICH, KIPAN, KIRP, LGG, LIHC, LUSC, OV, PAAD, PCPG, PRAD, READ, STAD, and UVM was associated with significant positive correlations between the three scores of ESTIMATEScore, ImmuneScore, and StromalScore (Figure 7D). The study also showed that *P4HA3* was significantly negatively correlated with nine different cancer-related functional statuses in GBM and UM, respectively (Figure 7E). Furthermore, as seen in the correlation heat map, *P4HA3* was closely associated with a variety of immune cells such as CD8^+^ T cells, CD4^+^ T cells, B cells, neutrophils, myeloid dendritic cells, macrophages, cancer-associated fibroblast (CAF), endothelial cells, and Hematopoietic stem cells in pan-cancer (Figure 8A). Thus, *P4HA3* is closely associated with tumor-associated immune cells and functional status.

### Correlation analysis of *P4HA3* and tumor stemness index

The analysis showed that *P4HA3* was significantly positively correlated with different tumor stemness indices such as DMPss (Figure 8B), DNAss (Figure 8C), ENHss (Figure 8D), and EREG-METHss (Figure 8E) in KIPAN, PRAD, GBM, LGG, LUAD, KIRC, KIRP, and SARC. And significant negative correlations were found in LIHC, LUSC, BLCA, STAD, STES, BRCA, COAD, and READ. The results of this analysis suggest that *P4HA3* is associated with tumor stemness.

### Analysis of *P4HA3* associated with mutation, methylation, CNV, TMB, MSI, MMR gene, and DNA methyltransferase

**FIGURE 9:**
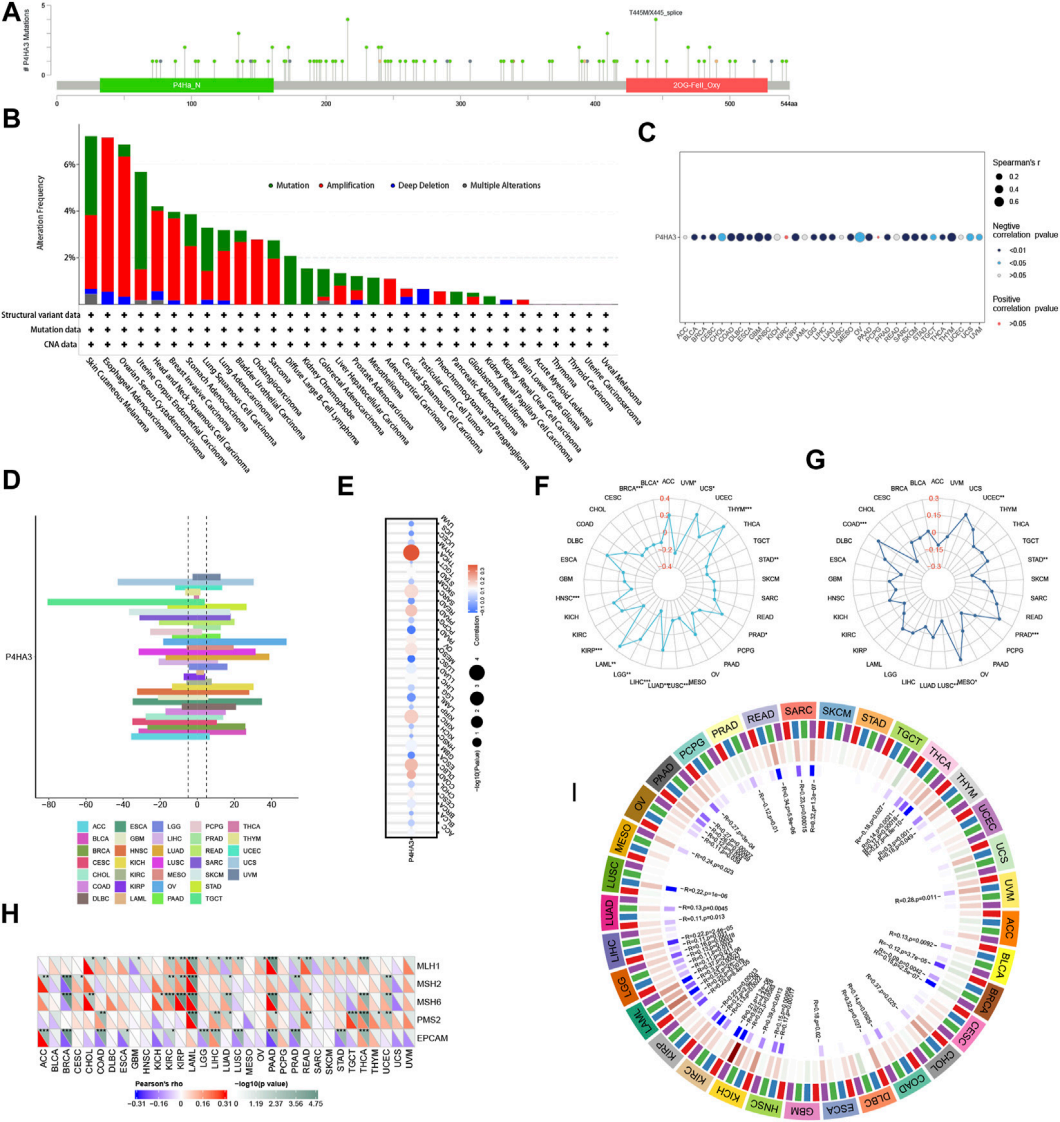
**(A)** Mutation sites of *P4HA3* and **(B)** The mutation frequency and corresponding mutation types of *P4HA3* in different cancers. **(C)** The Relationship of *P4HA3* expression with methylation. **(D)** CNV expression of *P4HA3* in human pan-cancer. **(E)** Correlation analysis of *P4HA3* CNV expression with *P4HA3* mRNA expression. The Relationship of *P4HA3* expression with **(F)** TMB and **(G)** MSI. Figure 10: Correlation analysis of *P4HA3* expression with **(H)** MMR genes in human pan-cancer (*p < 0.05, **p < 0.01, ***p < 0.001) and **(I)** DNA methyltransferases (Red represents DNMAT1, blue represents DNMT2, green represents DNMT3A, and purple represents DNMT3B).

The mutation sites of *P4HA3* are shown in Figure 9A. The mutation type was predominantly Amplification, with the highest mutation frequency of *P4HA3* in Esophageal Adenocarcinoma (Figure 9B). *P4HA3* was significantly negatively correlated with methylation levels in 25 tumors (Figure 9C). *P4HA3* had the highest CNV expression levels in TGCT and OV (Figure 9D). The correlation analysis showed that *P4HA3* and CNV expression levels were positively correlated in 26 tumors and negatively correlated in seven tumors (Figure 9E). As shown in Figure 9F, *P4HA3* was significantly and positively correlated with TMB in UCS, THYM, PRAD, LUAD, LGG, and LAML. And it was significantly negatively correlated with TMB in BRCA, BLCA, UVM, LUSC, LIHC, KIRP, and HNSC. As shown in Figure 9G, *P4HA3* was significantly and positively correlated with MSI in UCIEC, PRAD, MESO, COAD, and STAD. And it was significantly negatively correlated with MSI in STAD and LUSC. In addition, we analyzed the correlation between *P4HA3* and the level of MMR gene mutations. The results showed that the expression of *P4HA3* in KIRC, KIRP, LAML, LIHC, READ, THCA, and UCEC was significantly correlated with the mutation levels of five MMR genes (Figure 9H). Finally, we analyzed the correlation between *P4HA3* and the four DNA methyltransferases. The results showed that the expression level of *P4HA3* was significantly correlated with at least one DNA methyltransferase in other tumors except for SKCM, STAD, UCEC, UCS, ACC, CESC, GBM, and PCPG (Figure 9I).

## The results of GSEA

**FIGURE 10:**
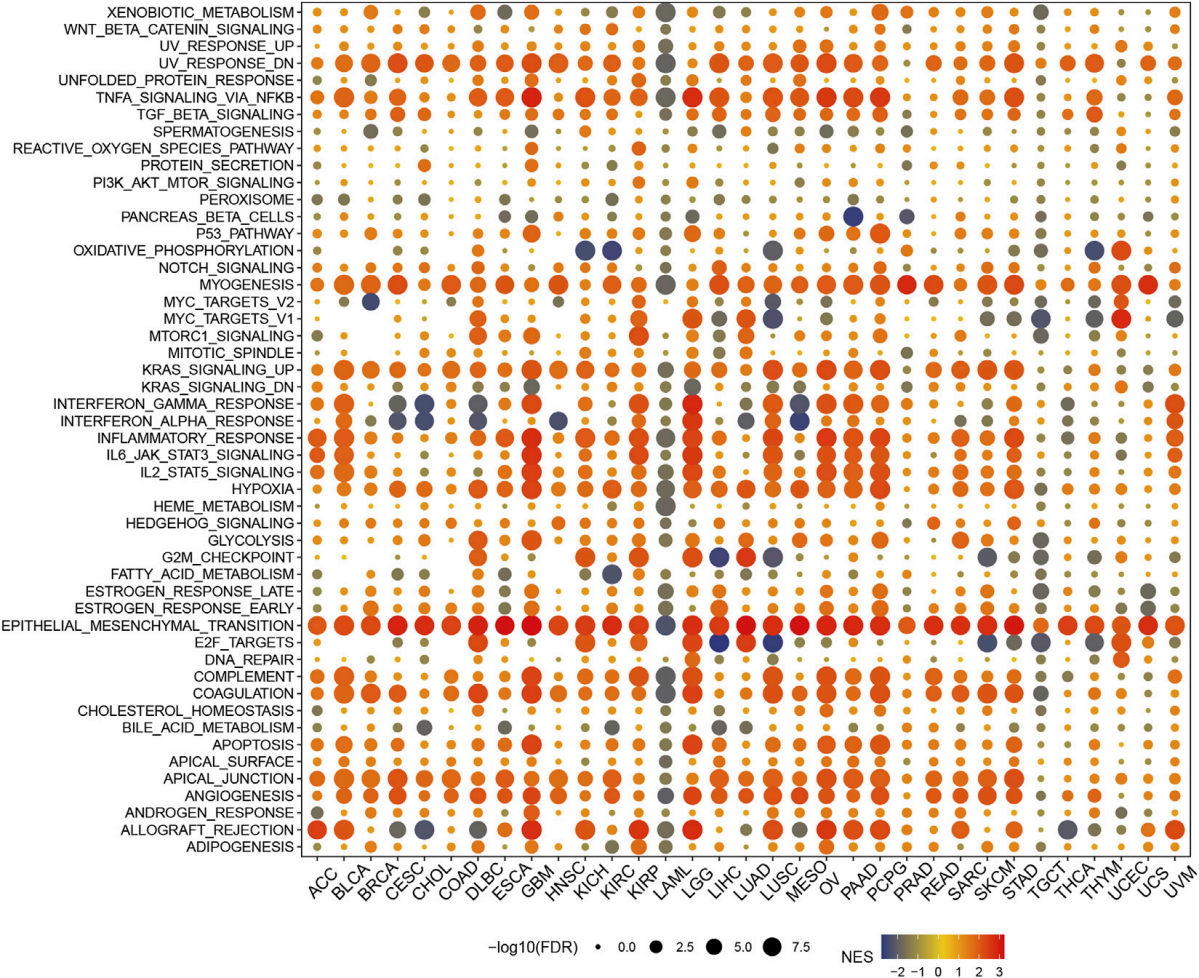
The hallmarks gene set enrichment analysis (GSEA) of *P4HA3* in pan-cancer. The circle size represents each cancer enrichment item’s false discovery rate (FDR) value, and the color represents the normalized enrichment score (NES) of each enrichment item.

The results of the GSEA analysis, as shown in Figure 10, showed that *P4HA3* showed a significant positive correlation with UV response dn, TNF-alpha signaling *via* NFkB, myogenesis, KRAS signaling up, inflammatory response, il6 jak stat3 signaling, hypoxia, epithelial-mesenchymal transition, apical junction, and angiogenesis in most tumors. The enrichment analysis results suggest that *P4HA3* is associated with multiple immune-related signaling pathways in tumors.

### Sensitivity analysis of *P4HA3* and drugs

**FIGURE 11:**
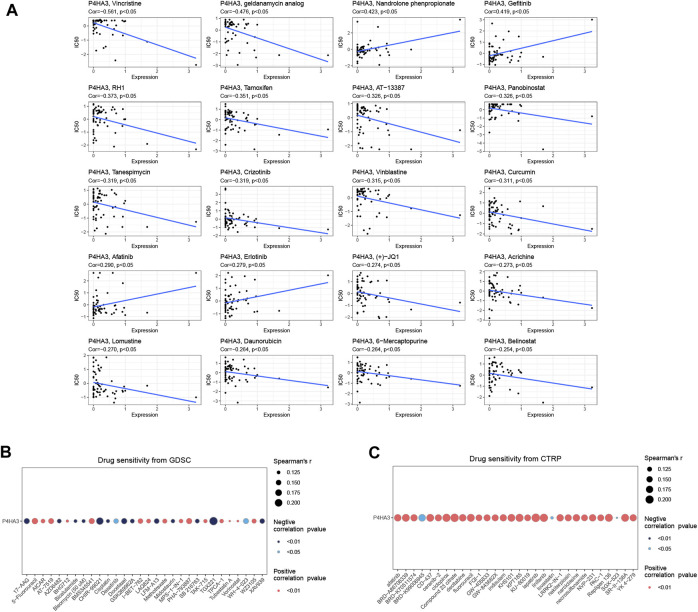
Drug sensitivity analysis. **(A)** In the CellMiner database, *P4HA3* was shown to be closely associated with 20 drugs. **(B)** A Spearman association between *P4HA3* and 30 drugs was performed in the GDSC database. **(C)** A Spearman association between *P4HA3* and 30 drugs was performed in the CTRP database.

In the CellMiner database, the expression of *P4HA3* was correlated with Vincristine, geldanamycin analog, RH1, Tamoxifen, AT-13387, Panobinostat, Tanespimycin, Crizotinib, Vinblastine, Curcumin (+)-JQ1, Acrichine, Lomustine, Daunorubicin, 6-Mercaptopurine, and Belinosta1 were significantly and negatively correlated with 16 drugs (Figure 11A). In the GDSC database (Figure 11B) and the CTRP database (Figure 11C), *P4HA3* was significantly positively correlated with various drugs.

## Discussion

Pan-cancer bioinformatics analysis can help us to understand the gene expression profile and its correlation with genetic mutations, clinicopathology, and prognosis (Ju et al., 2020). Besides, we can also use it to explore the potential mechanisms of gene roles in tumor immunology. Therefore, in this study, we systematically analyzed the expression of *P4HA3* in various cancers. We found that *P4HA3* was highly expressed in 16 tumors, including BRCA, CHOL, COAD, DLBC, ESCA, GBM, HNSC, LUAD, KIRC, LAML, LUSC, PAAD, PCPG, READ, STAD, and THYM. Combined with the Kaplan-Meier survival analysis results, we found that overexpression of *P4HA3* in BRCA, COAD, GBM, HNSC, KIRC, LUSC, READ, and STAD was associated with poor prognosis in patients with these cancers. *P4HA3* may serve as a promising biomarker for diagnosing and treating BRCA, COAD, GBM, HNSC, KIRC, LUSC READ, and STAD. Also, we found that the expression level of *P4HA3* was closely associated with OS, DSS, DFI, and PFI in patients with multiple cancers, especially in PAAD, KIRP, and CESC. In addition, we analyzed the relationship between the expression of *P4HA3* and the clinicopathological stage of cancer. It was found that the level of *P4HA3* expression was higher in stage IV than in phase I in BLCA, COAD, ESCA, KIRP, READ, and STAD. This result suggests that the high level of *P4HA3* expression correlates with the malignancy of these cancers.

Previous studies have found that collagen increases anti-programmed cell death protein 1 (PD-1)/PD-L1 resistance by depleting CD8^+^ T cells, which is associated with tumor immunosuppression and drug resistance (Chakravarthy et al., 2018; Mariathasan et al., 2018; Peng et al., 2020). Because of the above findings, we analyzed the relationship between *P4HA3* and immune regulatory genes and checkpoint genes. We found that *P4HA3* expression levels positively correlated with immune regulatory genes and checkpoint genes in most tumors. In addition, there is growing evidence that understanding tumor immune cell infiltration may assist in optimizing antitumor immunotherapy (Bai et al., 2020; Guo et al., 2020). Therefore, in this study, we also evaluated the correlation between *P4HA3* expression and tumor immune cell infiltration levels. The results suggest that *P4HA3* is associated with multiple immune cells in most cancers. Among them, the CD8^+^ T cell infiltration level was negatively correlated with the level of *P4HA3* expression in most tumors. In addition, we found that macrophage expression in various tumors showed the strongest positive correlation with *P4HA3* expression. A series of previous studies have also demonstrated that tumor-associated macrophages (TAMs) play an immunosuppressive role in tumor development and are associated with poor tumor prognosis (Kersten et al., 2022). TAMs can promote tumor growth through the production of collagen (Qiu et al., 2019). Notably, in this study, we also found a significant positive correlation between the degree of CAFs infiltration and *P4HA3* expression in pan-cancer. CAFs are a central component of TME and significantly affect cancer progression, metastasis, treatment, and prognosis (Lavie et al., 2022). They are involved in carcinogenesis as an essential cell type in the ECM that allows collagen deposition and even leads to tumor fibrosis (Lambrechts et al., 2018). These results suggest that *P4HA3* may play an essential role in the tumor immune microenvironment.

It is understood that the effectiveness of ICIs is mainly influenced by TMB and MSI (Postow et al., 2015; Dudley et al., 2016). Moreover, many studies also suggest that TMB and MSI may be potential predictive markers associated with tumor immunotherapy response and drug resistance (Yarchoan et al., 2017; Hellmann et al., 2018; Chae et al., 2019; Zhao et al., 2019). MMR Gene mutation status can predict tumor development (Cerretelli et al., 2020). CNA has been recognized as an essential source of genetic variation. In addition, both TMB and CNA are good predictors of the efficacy of ICIs, and the higher the predictive accuracy of the two combined (Liu et al., 2019). Here, we will evaluate the correlation of *P4HA3* expression with tumor TMB, MSI, MMR Gene, CNA, and mutational status. Among them, elevated *P4HA3* expression levels upregulated TMB of UCS, THYM, PRAD, LUAD, LGG, and LAML, as well as MSI of UCEC, PRAD, MESO, COAD, and STAD. In human cancers, *P4HA3* expression was significantly associated with five MMR genes, especially in LAML and THCA. We found a *P4HA3* gene variation rate of 2.6% in all TCGA tumors, dominated by missense mutations. In addition, epigenetic modifications are one of the main reasons affecting the effectiveness of various tumor immunotherapies, such as ICIs and immune cell therapies (Zebley et al., 2020). DNA methylation modifications are known to be one of the most important and well-studied epigenetic modifications (Skvortsova et al., 2019). DNA methyltransferases (DNMT) catalyze DNA methylation and are key epigenetic targets for new drug development (Lyko and Brown, 2005). After analysis, *P4HA3* expression was positively correlated with DNA methyltransferases in most tumors, especially in LAML. Downregulation of C-P4H in lymphoma is associated with DNA methylation (Hatzimichael et al., 2012). In addition to the most intensively studied DNA methylation, RNA methylation modifications have been increasingly recognized as an essential element of epigenetics in recent years (Roundtree et al., 2017). RNA modifications are widely present in the fundamental biological processes required for cancer development and are closely associated with tumor progression, including N1-methyladenosine (M1A), 5-methylcytosine (M5C), and N6-methyladenosine (M6A) (Zhao et al., 2017). We found that *P4HA3* was significantly associated with m1A, m5c, and m6A-related genes in most tumors, especially m6A-related genes. For example, *P4HA3* expression was positively correlated with the expression of more than 20 m6A RNA modification regulators in LUAD, BRCA, COADREAD, PAAD, LIHC, OV, THCA, UCEC, KIPAN, UVM, KICH, and LAML. Many previous studies have shown that m6A-related genes are promising targets for cancer immunotherapy (Han et al., 2019; Guo et al., 2021; Zhao et al., 2021). In addition, abnormal m6A methylation can affect the expression levels of tumor target genes (Guo et al., 2021). In summary, *P4HA3* may be involved in tumor development by regulating genetic mutation status or epigenetic modifications. Its expression level can be used as a potential predictor to assess the efficacy of immunotherapy in cancer patients.

The ability of human stem cells (SCs) to self-renew and differentiate into mature cells is called stemness (Phi et al., 2018). Many studies have found that cancer stem cells (CSCs) are associated with tumor proliferation, metastasis, recurrence, and drug resistance (Ayob and Ramasamy, 2018; Kuşoğlu and Biray, 2019). Here, we found that *P4HA3* was correlated with the dryness index. These findings may help us identify new biomarkers that can predict tumor progression, guide more targeted treatment strategies, and predict prognosis. We performed functional enrichment analysis to understand the potential molecular functions of P4HA3 and related signaling pathways. The results suggest that *P4HA3* is closely associated with various biological processes. Among them, *P4HA3* was most significantly correlated with epithelial-mesenchymal transition. Many previous studies have shown that *P4HA3* promotes tumor cell growth, proliferation, and metastasis in HNSC by activating the EMT process (Wang et al., 2020). Targeting *P4HA3* inhibits EMT in colon cancer (Zhou et al., 2022a). TGF-β enables cancer cell invasion and metastasis and inhibits the anti-tumor activity of immune cells (Derynck et al., 2021). TGF-β stimulation increases *P4HA3* expression, which is associated with EMT in non-small cell lung cancer (Nakasuka et al., 2021). *P4HA3* reverses the inhibitory effect of COL6A6 on EMT in pituitary adenoma (PA) (Long et al., 2019). Reduced levels of *P4HA3* expression significantly inhibited the EMT process in colon cancer (Zhou et al., 2022b). Numerous studies have confirmed that EMT is closely related to tumor metastasis (Zhang et al., 2019; Gaballa et al., 2020; Cai et al., 2021). This evidence, combined with our current findings, further demonstrates that *P4HA3* may be involved in tumor progression in pan-cancer through multiple biological processes with EMT as the primary role. Finally, we also analyzed the relationship between *P4HA3* and drug sensitivity. Twenty drugs were found to be associated with *P4HA3*.

Our findings suggest that *P4HA3* is expressed at high levels in most cancers, which correlates with the spite of cancers and poor prognosis. We can also assess the effect of tumor immunotherapy, tumor immunosuppression, and drug resistance based on the expression of *P4HA3*. In addition, *P4HA3* can affect the infiltration of tumor immune cells and is likely to promote cancer progression through EMT. Therefore, *P4HA3* may serve as a promising biomarker for human cancer diagnosis, treatment, and prognosis and may predict the efficacy of anti-tumor immunotherapy response. This study may provide ideas for future researchers to study the role of *P4HA3* in human cancers.

Of course, there are some limitations to this study. For example, our study was limited to bioinformatics analysis, which remains to be further validated by basic experiments or clinical trials. In addition, although the present study identified that *P4HA3* might affect cancer by participating in some potential signaling pathways, the specific molecular biology of the mechanism of action is not yet clear. These need to be further investigated in depth in the future.

## Data Availability

The original contributions presented in the study are included in the article/Supplementary Material, further inquiries can be directed to the corresponding authors.
