# Corrigendum: Systematic pan-cancer analysis of the potential tumor diagnosis and prognosis biomarker P4HA3

**DOI:** 10.3389/fgene.2023.1204421

**Published:** 2023-05-23

**Authors:** Yinteng Wu, Bo Zhang, Juan Nong, Raquel Alarcon Rodriguez, Wenliang Guo, Ying Liu, Shijian Zhao, Ruqiong Wei

**Affiliations:** ^1^ Department of Orthopedic and Trauma Surgery, The First Affiliated Hospital of Guangxi Medical University, Nanning, Guangxi, China; ^2^ Department of Trauma Hand Surgery, The Second Nanning People’s Hospital, Nanning, Guangxi, China; ^3^ Department of Joint Surgery, The Second Nanning People’s Hospital, Nanning, Guangxi, China; ^4^ Faculty of Health Sciences, University of Almerìa, Almeria, Spain; ^5^ Department of Rehabilitation Medicine, Guigang City People’s Hospital, Guigang, China; ^6^ Department of Rehabilitation Medicine, The First Affiliated Hospital of Guangxi Medical University, Nanning, Guangxi, China; ^7^ Department of Cardiology, The Affiliated Cardiovascular Hospital of Kunming Medical University (Fuwai Yunnan Cardiovascular Hospital), Kunming, Yunnan, China

**Keywords:** prolyl 4-hydroxylase subunit alpha 3 (P4HA3), pan-cancer, the tumor microenvironment (TME), collagen, immunotherapy, epithelial-mesenchymal transition (EMT)

In the published article, there was an error in Figure 1D as published. The figure was misspelled during the rework process. The corrected Figure 1 and its caption are given below.

**FIGURE 1:**
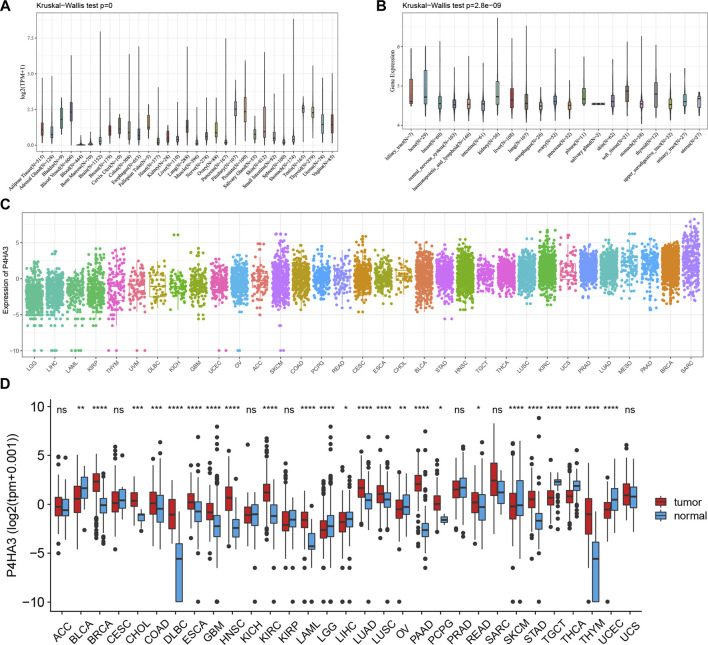
Expression of *P4HA3* in normal and tumor tissues. **(A)** *P4HA3* expression across 31 regular tissues and **(B)** 21 tumor cell lines. The mRNA expression landscape of *P4HA3* in **(C)** tumor tissue on TCGA database and **(D)** expression of *P4HA3* in normal and tumor tissues (*p < 0.05, **p < 0.01, and ***p < 0.001; ns: no significance). *p* values were based on the Wilcoxon rank sum test.

The authors apologize for this error and state that this does not change the scientific conclusions of the article in any way. The original article has been updated.

