# Supplementary figures and images for: Systematic pan-cancer analysis of the potential tumor diagnosis and prognosis biomarker P4HA3
Source: Front Genet. 2023 Mar 22;14:1045061. doi: 10.3389/fgene.2023.1045061 (PMC10073565; doi:10.3389/fgene.2023.1045061)

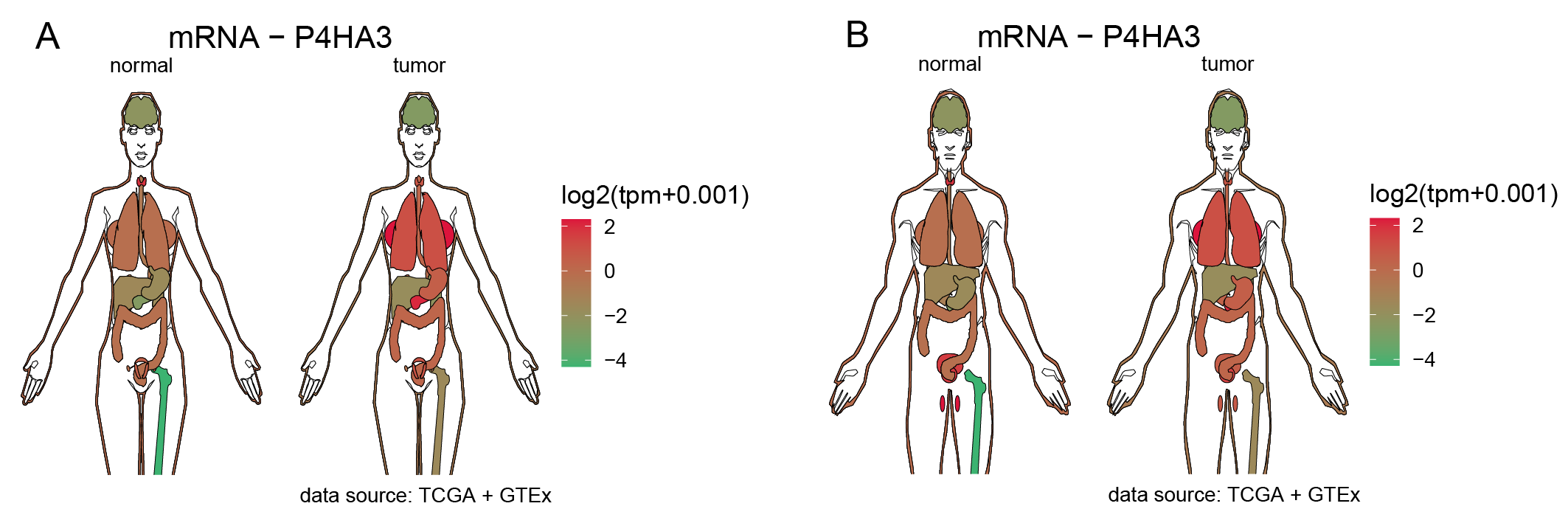

Supplement: Supplementary file 1 [file Image1.TIF]
